# Perspectives on disclosure of the dementia diagnosis among primary care physicians in Japan: a qualitatively driven mixed methods study

**DOI:** 10.1186/s12875-019-0964-1

**Published:** 2019-05-23

**Authors:** Michiko Abe, Shinji Tsunawaki, Masakazu Matsuda, Christine T. Cigolle, Michael D. Fetters, Machiko Inoue

**Affiliations:** 1 Department of Family and Community Medicine, Hamamatsu University School of Medicine, 1-20-1 Handayama, Higashi-ku, Hamamatsu, 431-3192 Japan; 2 Kikugawa Family Medicine Center, Kikugawa, Japan; 3 Shizuoka Family Medicine Program, Hamamatsu, Japan; 4 Department of Family Medicine, University of Michigan, Ann Arbor, MI USA; 5 Geriatric Research, Education and Clinical Center (GRECC), VA Ann Arbor Healthcare System, Ann Arbor, MI USA; 6 Department of Internal Medicine, University of Michigan, Ann Arbor, MI USA; 7 The School of Health Humanities, Peking University Health Science Center, Beijing, China

**Keywords:** Dementia, Disclosure, Japan, Primary care physicians, Qualitatively driven mixed methods study, Rural-urban comparison

## Abstract

**Background:**

The number of dementia patients in Japan is projected to reach seven million by 2025. While modern ethicists have largely reached the conclusion that full disclosure of dementia serves the best interest of patient, the implications of disclosure of a dementia diagnosis remains an underexplored area of research in Japan. The purpose of this study was to explore primary care physicians’ perspectives relative to the practice of disclosure of the dementia diagnosis.

**Methods:**

In this qualitatively driven mixed methods project, we conducted semi-structured interviews with 24 primary care physicians using purposeful sampling to identify rural and urban representation. All interview recordings were transcribed verbatim and analyzed thematically. The research team iteratively conducted discussions of the concepts as they emerged until reaching thematic saturation. The summary was distributed to the participants for member checking and we incorporated their feedback into the final analysis.

**Results:**

Of 24 participants, 12 practice in rural areas and 12 practice in urban/suburban areas. Participants’ attitudes varied in whether or not to disclose dementia diagnosis to the patients, and in the level of clarity of the name and the prognosis of the disease. Participants who were more comfortable in practicing disclosure were communicating collectively to the patients and their family members and those who were less comfortable practicing disclosure were concerned about patients’ feelings and had negative perceptions given the insidious progression of the disease.

**Conclusion:**

We found substantive individual differences in the approach to disclosure of the diagnosis of dementia and the level of comfort among primary care physicians. More dialogue about this issue and training to equip primary care physicians lacking confidence in their approach may be required.

**Electronic supplementary material:**

The online version of this article (10.1186/s12875-019-0964-1) contains supplementary material, which is available to authorized users.

## Background

With the increased aging of Japan’s population, the number of dementia patients in Japan is projected to reach seven million by 2025 [1]. As primary care physicians provide the bulk of clinical services to senior citizens on a daily basis, they serve as the front line of dementia care in the community [2]. In the early stage of dementia management, procedures for establishing the diagnosis of dementia have been standardized in a published guideline in Japan [3], yet how to deliver the diagnosis is not addressed in the guideline [3].

Since the 2000s, medical ethicists have largely concluded that disclosure of dementia serves the best interest of patients from the standpoint of protecting patients’ rights to self-determination [4, 5]. Consequently, ethical debates around disclosure have been shifting from “whether or not to disclose” to “how to disclose” [6]. Published literature [4–8] from around the world about disclosure of dementia recommends that disclosure should occur as a process rather than in a single visit and that disclosure should be individualized according to each patient’s unique circumstances.

However, Low et al. [9] in a systematic review of the literature drawn mainly from Europe, North America and Australia about practitioners’ practices and attitudes towards communicating a diagnosis of dementia found that only 34% of primary care physicians reported disclosing the diagnosis to patients. Previous studies on barriers to disclosure cite: concern about the negative psychological impact on patients [4, 6, 7, 9, 10]; the patient’s level of understanding of the information [4, 10]; feelings of stigma [6, 9, 11]; the futility of treatment [6, 7, 9, 10]; and lack of confidence in making the correct diagnosis [9, 12]. These reports suggest a gap between recommendations and what occurs in practice [7, 13]. Moreover, these guidelines and findings predominantly come from industrialized nations in Europe and North America. As such, they may not speak to primary care physicians’ practices of disclosing dementia in Asia, where healthcare systems and culture differ from those of Europe and the Americas [14, 15].

The implication of disclosing dementia remains an underexplored area of research in Japan [16]. The existing literature in Japan primarily addresses how disclosure should be conducted based on ethicist or specialist opinions [17, 18]. A survey of the general population in an urban city regarding disclosure of dementia reveals that the majority of respondents prefer to be told given a hypothetical diagnosis of dementia [19]. As primary care physicians’ experiences, beliefs, and practices on this issue have not been investigated in Japan, the purpose of this study was to explore rural and suburban/urban primary care physicians’ approaches and perspectives relative to the practice of disclosure of a dementia diagnosis.

## Methods

### Design and setting

We employed a qualitatively driven mixed methods design organized through conduct of semi-structured interviews with 24 primary care physicians, and rankings of their comfort disclosing dementia. A qualitatively driven design overall relies on an qualitative approach, but incorporates with a lesser emphasis quantitative data collection as well [20]. This study is part of a larger investigation conducting a rural-urban comparison of primary care physicians’ dementia care for multimorbid older adults in the U.S. and Japan. The Institutional Review Board of Hamamatsu University School of Medicine, the home institution of the investigators in Japan, approved this research on December 27th, 2016 (No.16–233). We obtained written informed consent from all participants.

### Data collection instrument

Interview questions addressed primary care physicians’ goals in managing multimorbid patients with dementia; their practices of diagnosing, disclosing, and managing dementia; their comfort level with disclosing on a scale from 1 to 10 followed by probing for their rationale; and available resources for dementia care in their working environment (Additional file 1). As this project is part of a larger US-Japan comparison study, the interview guide initially was developed in collaboration with U.S. investigators. For use in Japan, a professional service translated the interview guide into Japanese and two bilingual senior investigators (MI, MDF) reviewed the content and confirmed the language and substance of the inquiry to be natural and appropriate for the Japanese context.

### Recruitment, sampling and data collection procedures

We purposefully recruited practicing primary care physicians by e-mail, using maximum variation sampling to achieve a mixture of practice area, gender, age and years of clinical experience. We included physicians based in clinics as well as local hospitals because they were both considered providers of primary care services in Japan. In Japanese definition, medical institutions with less than 20 beds, which consist of 7.1% of all clinics (*shinryo-jo*), are called “clinic with beds (*yu-sho-shinryo-jo*),” and those with 20 or more beds are called “hospital (*byo-in*).” Hospitals with less than 100 beds consist of 35.7% of all the hospitals in Japan, and patients have unrestricted access to such small-sized local hospitals as well as clinics, as primary care centers in the Japanese healthcare system [21]. From August to October in 2017, the first author (MA) conducted interviews in person at physicians’ offices or using video conferencing. She is a researcher with the background of inter-cultural and medical communication (Master’s degree) and training in qualitative research. Prior to the interview, the participants had no relation with the interviewer. The interviewer explained the purpose of the research and her background as a qualitative researcher, that she was not a medical professional and that participants could share their experience without being judged or evaluated. Interviews lasted 60 to 150 min and were audio-recorded. Although the dementia care process was discussed broadly during the interview, this paper focuses on primary care physicians’ practices of disclosure of dementia diagnosis.

### Qualitative data analysis

After each interview, the interviewer created an interview summary addressing the three constructs of “context”, “content” and “concepts” to capture the major issues that were discussed during the interview. Data collection and analysis occurred iteratively. The research team discussed the emerging findings in regular team meetings. Additionally, all interview recordings were transcribed verbatim, and then analyzed thematically using MAXQDA Analytics Pro 12. The text was coded by the first author (MA) according to emerging categories about dementia care that the participants discussed, e.g., diagnosis/disclosure/management/end of life care. For each of the categories, we identified themes [22]. The main themes and sub-themes related to “disclosure” are presented in Table 1. Interviewing continued until reaching thematic saturation. For member checking, we distributed a summary of the project findings, a copy of each participants’ interview summary, and a request for feedback. All 24 participants replied with agreement with the distributed summary, and 22 participants provided additional comments that we incorporated into the findings.

**Table 1:** Themes and sub-themes related to disclosure of dementia by primary care physicians in Japan (2017)

Themes	Sub-themes
Meaning of dementia	Neutral
	Bad news
Practice of disclosing dementia	Target (to whom)
	Content (what)
	The way to deliver (how)
Reaction of patients/family	
Difficulties disclosing dementia	Need to take care of emotions or resistance of patient/family
	Limited time in outpatient clinic
	Lack of experience
	Lack of training
Factors related to high confidence in disclosing dementia	Positive image of dementia as illness (manageable)
	Positive attitudes/beliefs for disclosing dementia

### Mixed data analysis

To merge participant responses about their level of comfort with disclosing dementia, and the reasoning behind their rankings, we used joint display analysis, the process of linking the quantitative and qualitative findings in a table or matrix according to constructs held in common between both types of data [23]. The final step involves drawing interpretations or what mixed methods research methodologists term “metainferences” based on both types of data that we organized in a final column [20].

## Results

As illustrated in Table 2, a total of 24 primary care physicians participated in the interviews: 12 who practice in rural areas and 12 who practice in urban/suburban areas. The average years of participants’ experience in clinical practice was 15 (6–38) years (since graduating from medical school), with 10.5 [3–22] years in primary care practice. Seventeen (71%) of the participants were male and 7 (29%) were female. Eighteen (75%) practiced in clinics while six (25%) worked in primary care hospital settings. Five participants had worked in clinics in both urban and rural areas. In these cases, we asked them to speak primarily about their experiences in the environment leaving the strongest impression on them and to compare the two environments whenever appropriate. In cases where we experienced difficulty in identifying the region of practice, i.e., suburban versus rural, we categorized participants according to their self-descriptions.

**Table 2:** Characteristics of participants (2017) (*n* = 24)

	Rural (*n* = 12)	Urban/Suburban (n = 12)	Total (n = 24)
Years practicing as physician^a^	12.7 ± 5.9 (6–24)	17.4 ± 8.1 (9–38)	15.0 ± 7.4 (6–38)
Years practicing as PCP^a^	9.0 ± 6.4 (3–22)	11.9 ± 5.2 (5–22)	10.5 ± 5.9 (3–22)
Gender^b^			
Male	9 (75%)	8 (67%)	17 (71%)
Female	3 (25%)	4 (33%)	7 (29%)
Setting^b^			
Clinic	8 (67%)	10 (83%)	18 (75%)
Hospital	4 (33%)	2 (17%)	6 (25%)

^a^Average: ±SD, ^b^numbers (%)

### How to disclose the dementia diagnosis

**Fig. 1:**
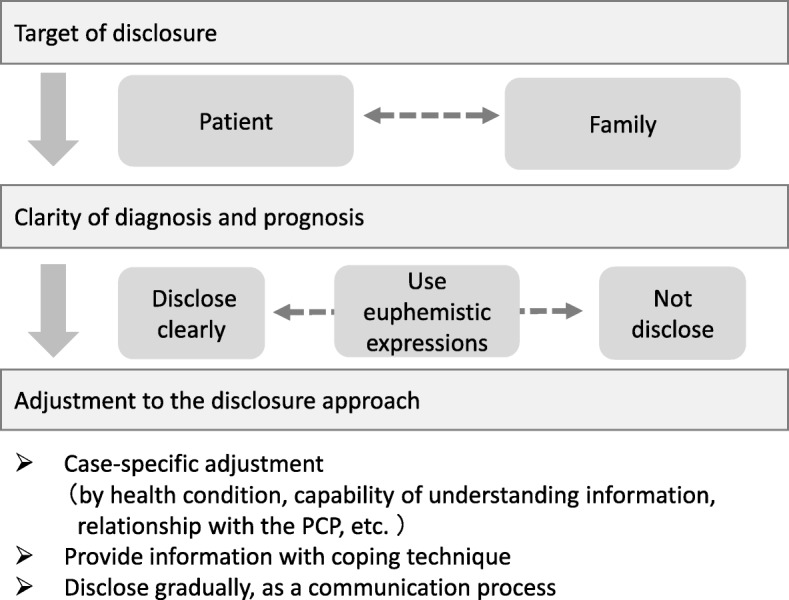
Three distinct themes where primary care physicians in Japan make choices about the disclosure of a dementia diagnosis (2017)

Participants’ attitudes about the disclosure of the dementia diagnosis varied (Fig. 1). There were three distinct themes where participants were making choices in practice: the target of disclosure; the clarity of diagnosis and prognosis; and adjustments made in the disclosure process. The following illustrates these themes in more detail.

### Target of disclosure

Participants were clearly divided in their beliefs about whether or not to disclose the dementia diagnosis to patients. Several of the primary care physicians willing to disclose dementia referred to the guidance of SPIKES (Setting, Perception, Invitation or information, Knowledge, Empathy, Summarize or strategize) [24] or similar methods for “breaking bad news” of a cancer diagnosis.

*As in the disclosure of a diagnosis of cancer, society is moving towards actively disclosing to patients rather than hiding the diagnosis from them. I am also trying to follow such a trend, and thus I basically do not hide diagnoses from my patients.* (Rural: male physician)

Four physicians stated that they only disclosed the diagnosis of dementia to patients’ family members. Some physicians also stated that, depending on the situation, they would discuss with patients’ family members as to how to share the diagnosis with the patients.

*I would probably disclose the diagnosis of dementia, definitely to patients’ family members, but almost certainly not to patients themselves.* (Urban: male physician)

*In cases I cannot decide, I first disclose to their family members and ask what they think, ….how should I tell the diagnosis to the patient.* (Rural: male physician)

One reason that disclosure of the diagnosis is often made to patients’ family members is that the process of diagnosing dementia tends to be initiated when patients’ family members raise a concern with the physician. It is often the family members who are troubled with and suffer from the patients’ symptoms of dementia and need care.

*Family members can be stressed out by thinking of the patient’s problem like wandering may continue forever. So, explaining the situation and prognosis, and offering concrete support to them is very important.* (Urban: female physician)

Another reason cited was the nature of the disease and how patients’ cognitive abilities naturally decline. Therefore, all participants felt to be essential to involve patients’ family members’ in supporting the current living situation of patients and in decision-making processes that concern medical and nursing care of patients.

When participants expressed hesitation about disclosing the diagnosis to their patients, their language evoked negative connotations such as “bad news” and “stigma”, and they expressed concerns about the potentially negative psychological impact of disclosure on patients.

*Current Japanese society has a sort of “stigma” associated with dementia so I should understand the context, the social meaning of making the diagnosis of “dementia,” how it will affect the individual. Such considerations make me think that “disclosure” is a difficult task.* (Urban, male physician)

### Clarity in the name and prognosis of the disease

Even among physicians who reported disclosing dementia directly to patients, there were differences in the degree of clarity in naming the disease and in describing its prognosis. The following statement was provided by a physician who reported that he fully gives the name and prognosis of the disease to his patients:

*In concrete terms, I tell my patients, right when the diagnosis is confirmed, that the average life expectancy ranges from five to seven years .... in the end, most patients die either from infections or from poor oral intake. I say such things in a matter-of-fact way.* (Rural: male physician)

In contrast, some participants used euphemisms rather than the word “dementia” at the time of diagnosis or disclosure. Examples include “forgetfulness (*mono-wasure*)”, so as not to pathologize the patient’s condition, or “fuzzy-headed (*boketa*)” and “senile (*chi-ho*)”, colloquialisms familiar to the local community.

With regard to prognosis, we found a tendency among physicians with many years of experience to not disclose fully at the time of the initial disclosure of the diagnosis. They cited concerns about causing patients to have anxiety while they were still able to comprehend such information. They also stated that dementia patients may live up to 10 years and that providing an accurate prognosis is difficult.

*I rarely touch upon the issues pertaining to prognosis.… Talking about such things could stir up anxiety in patients. I basically would not mention it, at least at the beginning.* (Rural: female physician)

### Adjustment in communication for disclosing dementia

The participants talked about a variety of efforts to communicate disclosure acceptably to patients and family members. The examples they gave included: adjusting the communication approach depending on the severity of dementia; being more sensitive for patients at an early stage of dementia; disclosing the diagnosis along with advice including recommendations for medication, how family members should care for patients, and information regarding long-term care services or care resources in the local communities; and disclosing the diagnosis and prognosis gradually. One physician contrasted disclosure of dementia and cancer. He described disclosure of dementia not as a discrete instance but as part of communication embedded in a continuous process of care.

*It is not like saying, “you have cancer,” …. I have my patients’ family members understand that their parent(s) have dementia and then continue to follow their situation. In time, when appropriate, I tell them that there may be such and such symptoms in the future. I might also explain to them what they could do when those symptoms appear.* (Rural: male physician)

### Comfort level in disclosing methods

The results of mixed data analysis about participants’ level of comfort with disclosing dementia, and the reasoning behind their rankings are presented in Table 3. We interpreted the characteristics of physicians based on the comfort level: highly comfortable (8 to 10); moderately comfortable (5 to 7); low comfort level (2 to 4); and no response as follows.

**Table 3:** Participating Japanese primary care physicians’ comfort with disclosing dementia to patients, explanations for their choices and comparison of across the four groups (2017)

Level of comfort	Quote	Interpretation
High (8~10) *n* = 9	“They come to see me because they want to know. So I would avoid telling them anything vague.” (Urban, female) “In the case of a patient whose cognitive level is low, I will talk to the family members rather than the patient.” (Rural, male) “I try not to do it by myself as much as possible…I usually ask a social worker, nurse or care manager to join.” (Rural, male)	-No hesitation for disclosure -Collective approach to patients and family members -Seek cooperation of other professionals
Moderate (5~7) n = 9	“I think dementia patients have fear about the future. I am not sure if I am taking care of such feelings well enough.” (Urban, male) “I don’t know at all if my way of disclosing dementia is appropriate. I have never received feedback from anyone.” (Urban, female)	-Concerns about patients’ feelings -Not sure about appropriate ways of disclosing dementia
Low (2~4) *n* = 4	“Because it is an incurable disease, I don’t have confidence to tell the diagnosis to patients.” (Urban, male) “The measure of whether or not to disclose to patients is rather unclear in my thinking”. (Rural, male) “I have been accused by my patient, ‘Don’t you dare treat me like a demented man!’” (Urban, female)	-Negative perceptions of dementia -Hesitation for disclosure -Feeling somewhat “guilty” for their own attitudes
No responders n = 2	“[On remote islands] People take it as one event life’s course. They would say, forgetfulness is just a natural aging process, it’s OK.” (Rural, female) “I don’t’ see any merit to telling the dementia diagnosis to patients.” (Urban, male)	-No need or benefit to disclosing dementia

#### Highly comfortable group

The highly comfortable group have a stance of disclosing dementia diagnosis collectively to the patient and the family members. If disclosure to the patient does not occur easily, they quickly shift the target of disclosure to family members. Some physicians in this group seek cooperation from other professionals in disclosing to the patient.

#### Moderately comfortable group

The moderately comfortable group reported focusing more on patients and showing concern about patients’ feeling when disclosing a dementia diagnosis. Many of them explained that they are practicing disclosure according to recommendations in the literature but that they find it difficult to evaluate the appropriateness of recommendations for their own practice.

#### Low comfort level group

The low comfort level group tend to emphasize negative aspects of dementia such as it being “an incurable disease,” “hopeless,” and “stigmatized.” Some physicians in this group reported experiences of patients reacting emotionally to disclosure of the dementia diagnosis. They tend to refrain from disclosing to their patients or only vaguely described their own standards for disclosure and some felt ‘guilt’ about their approach.

#### Non-responders

The fourth group, who did not respond with any level of comfort in disclosure, stated that they consider dementia as a natural process of aging. They do not see the need for or a situation when they should disclose dementia as a disease.

### Rural versus urban considerations

One pattern we observed related to practicing in extremely rural environments in remote islands. Here, some physicians find no need for disclosing dementia because the local community accepts dementia as changes due to aging. As there are few public care services in this environment, it lessens the opportunity to be asked to make a diagnosis of dementia.

*I would deliver the information as “disclosing bad news” when practicing in an urban area, but on an island, it is not like that. People there would say “they just forget,” and understand it as a natural process.* (Rural and Urban: female physician)

In addition, some physicians who were practicing in urban areas after previously working on a remote island described that the demand by family members for a clear diagnosis is usually stronger in urban compared to rural settings.

*[In the urban setting] it is patients’ family members who ask for a clear diagnosis or to identify dementia by testing. So, I communicate with them and pay attention to their life condition before seeking the diagnostic name or doing testing.* (Rural and Urban: male physician)

## Discussion

The present study investigates qualitatively how primary care physicians in Japan approach disclosure of the diagnosis of dementia to patients and family members. We found substantive individual differences in the approach to disclosure of the diagnosis of dementia and the level of comfort among primary care physicians. For a number of participants, the target of disclosure of dementia was collectively the patient and his/her family members. While recent ethical discussions conclude that disclosure of the diagnosis serves the best interest of patients [4, 5, 7, 25], our participants always included family members when disclosing dementia. The findings of this study illuminate how primary care physicians in Japan consider the cooperation and well-being of patients’ family members as crucial factors in maintaining dementia patients’ quality of life. It further speaks to a view of the family’s centrality in decision making [26]. From a Western ethical perspective, this may be seen as potentially undermining patient autonomy, though from a Japanese perspective, the emphasis may be seen as buttressing harmony of the patient’s existence within the family. As in other countries in East Asia, this tendency towards collectivism has arguably been influenced by Confucianism [27, 28]. There are clearly different cultural values and meanings relative to terminal illness [29, 30] and end-of-life-care in Japan [31] than in Western views. Approaches to dementia disclosure in other Asian cultures seems ripe for further investigation as well.

When focusing on the disclosure to the patient themselves, our participants share concerns which are common with previous studies such as the negative psychological impact on patients [4, 6, 7, 9, 10]; the degree patients understand provided information [4, 10]; and the stigma associated with the disease [6, 9, 11]. Physicians less comfortable with disclosing dementia emphasized barriers and concerns in telling the diagnosis to the patient. In a study that developed tools to support diagnostic delivery of dementia, Bennett et al. [32] discuss considering the emotional journey of clinicians. To achieve effective delivery of information, they opine physicians need to be aware of their own anxiety and travel through patients’ emotional journey at the same time [32]. Most of the participants seem to lack opportunities to share their concerns about this topic with colleagues, so creating such opportunities could help them become more self-reflective [10, 33] about their approaches. Physicians with motivation to change or lacking confidence could benefit from training focusing on communicating the diagnosis of dementia [6, 9–11, 34, 35].

About half of participants were unsure if their approach to disclosure was appropriate, mentioning they had had no training in such communication. Other participants referred to SPIKES [24], which in general provides a framework for “breaking bad news” including cancer recurrence. This suggests that communication guidance for dementia patients may be considered similar to cancer patients. However, while both are incurable diseases, dementia is characterized by more severe cognitive issues as the severity progress. Tuffrey-Wijne et al. [8] provides a thoughtful discussion about how the knowledge of the past, present, and future gradually shrinks when people have dementia. Thus, “what” and “how much” they should be told about their diagnosis needs careful consideration. This type of in-depth knowledge of communication that incorporates consideration of the characteristics of dementia can be valuable, particularly for primary care physicians who provide a wide range of clinical services and dementia care in their clinical practice.

Despite reports in other countries of insufficient care services [6, 7, 9, 10] or a lack of confidence in having the correct diagnosis [9, 12] as key obstacles to disclosure, these issues did not arise in the current study. We consider the reason for this result to be the national health system’s long-term care services in each region; patients will have health care and nursing care support based on the diagnosis and care-service contract, regardless of disclosure status to patients themselves [15]. When primary care physicians observe cognitive issues in a patient, their attention seems more likely to shift to interventions for supporting the patient’s and family’s living environment by collaborating with multi-professional staff to alleviate care burdens, rather than accurately diagnosing the subtype of dementia. Such a focus ostensibly could lead to insufficient investigation in primary care and the under diagnosis or misdiagnosis of treatable dementia or preclude having the most accurate prognostic information. Developing primary care physicians’ ability and confidence for making the correct diagnosis of dementia, communicating about dementia seem to be areas of importance for primary care physicians in Japan.

In our urban-rural comparison, we found a tendency among some physicians when working on remote islands to dismiss the need to disclose a dementia diagnosis because of tolerant attitudes of the community towards dementia and lack of public care services. Otherwise, the differences in attitudes about disclosing dementia varied more based on primary care physicians’ individual beliefs than their practice environment.

A limitation of this study is that the research participants were relatively young physicians and members of the Japan Primary Care Association. Most of our participants were family physicians certified by the Japan Primary Care Association, which only started in 2006 and remains a relatively new organization [36]. Hence these findings may not apply to more senior general practitioners who never completed systematic training in primary care experienced by younger physicians such as in this study. It remains unclear if the years of clinical practice or the environment in which physicians work affect physicians’ perspectives on disclosure. Future research using a survey among a larger population could delineate associations between physician characteristics, the work environment, and physicians’ attitudes and anxiety about disclosure.

## Conclusions

We found substantive individual differences in the approach to disclosure of the diagnosis of dementia and the level of comfort among primary care physicians in Japan. A number of participants practice dementia disclosure collectively to the patient and family members. Some were unsure about the appropriate ways to approach to patients themselves. These findings suggest a need for more dialogue about this issue and training to equip primary care physicians lacking confidence in their approach and motivated to disclose dementia to their patients.

## Additional file

Additional file 1: Interview Guide. (DOCX 22 kb)

## Abbreviations

SPIKES: Setting, Perception, Invitation or information, Knowledge, Empathy, Summarize or strategize

## References

[1] Government of Japan. 2017 White Paper of Aging Society (in Japanese) https://www8.cao.go.jp/kourei/whitepaper/w-2017/html/gaiyou/index. Accessed 18 Apr 2019.

[2] Health and Global Policy Institute, McCann Global HealthInstitute. Social Prescription for Dementia (in Japanese). 2017. https://hgpi.org/research/747.html. Accessed 18 Apr 2019.

[3] Societas Neurologica Japonica. Management Guideline of Dementia Disease 2017 (in Japanese). Igaku-Shoin Ltd.; 2017. https://www.neurology-jp.org/guidelinem/nintisyo_2017.html. Accessed 18 Apr 2019.

[4] Cornett PF, Hall JR (2008). Issues in disclosing a diagnosis of dementia. Arch Clin Neuropsychol.

[5] Fisk JD, Beattie BL, Donnelly M, Byszewski A, Molnar FJ (2007). Disclosure of the diagnosis of dementia. Alzhheimer’s & Dementia.

[6] Werner P, Karnieli-Miller O, Eidelman S (2013). Current knowledge and future directions about the disclosure of dementia: a systematic review of the first decade of the 21st century. Alzheimers Dement.

[7] Carpenter B, Dave J (2004). Disclosing a dementia diagnosis. The Gerontologist.

[8] Tuffrey-Wijne I, Watchman K (2015). Breaking bad news to people with learning disabilities and dementia. Learn Disabil Pract.

[9] Low LF, McGrath M, Swaffar K, Brodaty H (2018). Communicating diagnosis of dementia: a systematic mixed studies review of attitudes and practices of health practitioners. Dementia(London)..

[10] van den Dungen P, van Kuijk L, van Marwijk H, van der Wouden J, Moll van Charante E, van der Horst H (2014). Preferences regarding disclosure of a diagnosis of dementia: a systematic review. Int Psychogeriatr.

[11] Gove D, Downs M, Vernooij-Danssen M, Small N (2016). Stigma an GP's perception of dementia. Aging Ment Health.

[12] Phillips J, Pond CD, Paterson NE, Howell C, Shell A, Stocks NP (2012). Difficulties in disclosing the diagnosis of dementia: a qualitative study in general practice. Br J Gen Pract.

[13] Lecouturier J, Bamford C, Hughes JC, Francis JJ, Foy R, Johnston M (2008). Appropriate disclosure of a diagnosis of dementia: identifying the key behaviours of 'best practice'. BMC Health Serv Res.

[14] Betsch C, Böhm R, Airhihenbuwa CO, Butler R, Chapman GB, Chapman GB (2016). Improving medical decision making and health promotion through culture-sensitive health communication: an agenda for science and practice. Med Decis Mak.

[15] Nakanishi M, Nakashima T (2014). Features of the Japanese national dementia strategy in comparison with international dementia policies: how should a national dementia policy interact with the public health- and social-care systems?. Alzheimers Dement.

[16] Hamahashi K, Andou M, Miyabayashi Y (2016). A literary review of the actual status and problems of dementia notification. Kawasaki medical welfare journal.

[17] Imai Y (2011). Diagnostic disclosure to dementia patient in order to start medical treatment (in Japanese). J therapy.

[18] Yamaguchi N (2006). A study about diagnostic disclosure to dementia patients (in Japanese). Public Heath.

[19] Umegaki H, Onishi J, Suzuki Y, Endo H, Iguchi A (2007). Attitudes toward disclosing the diagnosis of dementia in Japan. Int Psychogeriatr.

[20] Johnson RB, Onwuegbuzie AJ, Turner LA (2007). Toward a definition of mixed methods research. J Mixed Methods Res.

[21] Ministry of Health, Labour and Welfare. Research of medical institutions (in Japanese). 2017. https://www.mhlw.go.jp/toukei/saikin/hw/iryosd/17/dl/02sisetu29-1.pdf. Accessed 10 May 2019.

[22] Kuckartz U. Three major methods in qualitative text analysis. Qualitative text analysis (Japanese translation). Tokyo: Shin-yo-sha; 2018. p. 91–160.

[23] Guetterman TC, Fetters MD, Creswell JW (2015). Integrating quantitative and qualitative results in health science mixed methods research through joint displays. Ann Fam Med.

[24] Kaplan M (2010). SPIKES: a framework for breaking bad news to patients with cancer. Clin J Oncol Nurs.

[25] Karnieli-Miller O, Werner P, Aharon-Peretz A, Eidelman S (2007). Dilemmas in the (un)veiling of the diagnosis of Alzheimer’s disease. Patient education and counseling.

[26] Fetters MD (1998). The family in medical decision making: Japanese perspectives. J Clin Ethics.

[27] Fan R. Rights or virtues? Towards a reconstructionist Confucian bioethics. In: Qiu R, editor. Bioethics: Asian perspectives. Philosophy and medicine, vol. 80: PHME; 2004. p. 57–68.

[28] Yu K-P. The Confucian alternative to the individual-oriented model of informed consent: family and beyond. In: Fan R, editor. Family-oriented informed consent philosophy and medicine, vol. 121: PHME; 2015. p. 93–106.

[29] Elwyn TS, Fetters MD, Gorenflo W, Tsuda T (1998). Cancer disclosure in Japan: historical comparisons, current practices. Soc Sci Med.

[30] Elwyn TS, Fetters MD, Sasaki H, Tsuda T (2002). Responsibility and cancer disclosure in Japan. Soc Sci Med.

[31] Fetters M, Danis M. Death with dignity: cardiopulmonary resuscitation in the United States and Japan. In: Engelhardt HT, Rasmussen LM, editors. Bioethics and moral content: National Traditions of health care morality. Philosophy and medicine, vol. 74: PHME; 2002. p. 145–63.

[32] Bennett CE, De Boos D, Moghaddam NG. Developing a tool to support diagnostic delivery of dementia. Dementia(London). 2018:1–27. 10.1177/1471301217750936 29378425

[33] Novack DH, Suchman AL, Clark W, Epstein RM, Najberg E, Kaplan C (1997). Calibrating the physician - personal awareness and effective patient care. JAMA..

[34] Kaduszkiewicz H, Bachmann C, van den Bussche H (2008). Telling “the truth” in dementia--do attitude and apprach of general practitioners and specialists differ?. Patient Educ Couns.

[35] Vince A, Clark C, Wolverson EL (2017). The meaning and experience of well being in dementia for psychiatrists involved in diagnositic disclosure: a qualitative study. Int Psychogeriatr.

[36] Takemura Y (2011). Past, present, and future of family medicine training program system (in Japanese). J General and Family Medicine.

